# Efficacy of tazemetostat in combination with R-CHOP in elderly patients newly diagnosed with diffuse large B cell lymphoma: results of the EpiRCHOP phase II study of the LYSA

**DOI:** 10.1016/j.eclinm.2025.103157

**Published:** 2025-03-18

**Authors:** Clémentine Sarkozy, Thierry Jo Molina, Sydney Dubois, Cédric Portugues, Elodie Bohers, Loic Ysebaert, Roch Houot, Gian Matteo Pica, Philippe Ruminy, Charles Herbaux, Thomas Gastinne, Catherine Thieblemont, Corinne Haioun, Stéphanie Guidez, Christophe Bonnet, Gilles Crochet, Liana Veresezan, Sylvain Choquet, Emmanuel Bachy, Fabrice Jardin, Franck Morschhauser, Vincent Ribrag

**Affiliations:** a Service d'hématologie, Institut Curie, Paris, France; b Laboratoire d'imagerie translationnelle en oncologie, U1288, Université Versailles Saint Quentin en Yveline, Saint Quentin en Yveline, France; c Service de pathologie, Necker Enfants Malades Hospital, Université Paris Cité, APHP, France; d Service d'hématologie, Centre Henri Becquerel, Rouen, France; e LYSARC, Pierre Bénite, France; f Inserm U1245, Normandie University, Centre Henri Becquerel, Rouen, France; g Service d'hématologie, IUC Toulouse-Oncopôle, Toulouse, France; h Service d'hématologie, CHU Rennes, Rennes, France; i Service d'hématologie, Centre Hospitalier de Chambéry, France; j Service d'hématologie, CHU Montpellier, Montpelliers, France; k Service d'hématologie, CHU Nantes, Nantes, France; l Service d'hématologie, Hôpital Saint Louis, APHP, France; m Service d'hématologie Lymphoide, Hôpital Henri Mondor, APHP, France; n Service d'hématologie, CHU de Poitiers, Poitiers, France; o Service d'hématologie, CHU de Liège, Liège, Belgium; p Hematology Department, CHU UCL Namur, Yvoir, Belgium; q Service de pathologie, Centre Henri Becquerel, Rouen, France; r Service d'hématologie, CHU Pitié Salpetrière, APHP, Paris, France; s Service d'hématologie, Centre Hospitalier Lyon Sud, Hospices Civiles de Lyon, Pierre Bénite, France; t Service d'hématologie, U918 Centre Henri Becquerel, Rouen, France; u Service d'hématologie, U1245 Centre Hospitalier RU de Lille, Lille, France; v Service d'hématologie, Institut Gustave Roussy, Université Paris-Saclay, INSERM U1170, Villejuif, France

**Keywords:** Diffuse large B cell lymphoma, EZH2, Phase II

## Abstract

**Background:**

In the phase I Epi-RCHOP study (NCT02889523), we reported that R-CHOP-tazemetostat was well tolerated with the recommended phase II dose, consistent with monotherapy.

**Methods:**

Phase II included newly diagnosed diffuse large B cell lymphoma patients aged 60–80 years who received six cycles of rituximab-CHOP (R-CHOP) with continuous tazemetostat (800 mg BID), plus two cycles of tazemetostat and rituximab (cycles 7 and 8), from July 31, 2020 to July 18, 2022. Primary endpoint was positron emission tomography complete metabolic response (CMR). Sample size was calculated with H0 of 70% and H1 assumption of 80%.

**Findings:**

The trial enrolled 122 patients: median age 70 (60–80), 90.2% with stage III–IV, and 73.8% with International Prognostic Index 3–5. Overall, 100 patients (82%) received eight cycles, while 22 had premature treatment discontinuation (PTD), including 12 during the first two cycles. Reasons for PTD were consent withdrawal (N = 10), adverse events (N = 6), death (N = 2), protocol deviation (N = 2), progressive disease (N = 1), and physician decision (N = 1). The median percentage of relative dose intensity of tazemetostat and R-CHOP exceeded 90%, but required a protocol amendment and reduction in vincristine dosage at 1 mg full dose. At the end of treatment or PTD, 92/122 patients (75.4%) achieved CMR, eight (6.6%) partial metabolic response, five (4.1%) progressive disease, two (1.6%) died (septic shock), and 15 (12.3%) were not evaluated. Sensitivity analysis, excluding ten non-evaluated patients who withdrew consent, showed CMR in 82.1%. After a median follow-up of 18.5 months (IQR: 15.4–21), estimated progression-free and overall survival at 18 months were 77.7% (95% CI: 67.5–85.1%) and 88.8% (95% CI: 79.9–93.9%), respectively.

**Interpretation:**

R-CHOP plus tazemetostat is feasible with a promising CMR in elderly DLBCL patients. Complementary biomarker studies are needed for a more personalized approach.

**Funding:**

This study was sponsored under a grant from 10.13039/501100014382Ipsen.

**Research in context**

**Evidence before this study**

In diffuse large B cell lymphoma (DLBCL), gain-of-function mutations in the histone methyltransferase *EZH2*, which are present in 15% of cases, and the loss-of-function abnormalities in the SWI/SNF complex induce an aberrant proliferative dependency on EZH2.^1^ Tazemetostat is an oral inhibitor of EZH2, approved for use in follicular lymphoma^2^ and activity in both *EZH2*-mutant and wild-type DLBCL.^3^ In DLBCL, elderly patients represent a challenging population with 30–40% of primary disease refractory to the standard of care rituximab-CHOP (R-CHOP), and potential safety issues in combination. We searched PubMed, with no restrictions on language or publication date, using the search terms “tazemetostat” and (“CHOP” OR “R-CHOP”) and did not find any studies reporting this combination.

**Added value of this study**

After establishing the recommended phase II dose in phase Ib of the trial, the phase II presented here enrolled patients aged 60–80 years with newly diagnosed DLBCL. For the first time, it reports data on the efficacy of the tazemetostat and R-CHOP combination.

**Implications of all the available evidence**

Results of this study suggest that the combination of tazemetostat plus R-CHOP is safe in the elderly DLBCL population. The relative dose intensity of R-CHOP was maintained, and the complete response rate in patients receiving the tazemetostat-R-CHOP combination is promising. Prolonged follow-up is needed to assess the potential role of biomarkers for this combination.

## Introduction

Diffuse large B-cell lymphoma (DLBCL) is the most common subtype of non-Hodgkin lymphoma (NHL) accounting for about 30% of all lymphoid neoplasms. Incidence increases with age, with around half of patients aged over 60 years and almost one-third over 75 years.^4^^,^^5^ In patients older than 60 years, DLBCL can be cured in 50–60% of cases following first-line treatment with the current standard of care, namely rituximab-CHOP (R-CHOP).^6^^,^^7^ However, 30–40% of patients present primary refractory DLBCL or early relapse disease associated with poor overall survival.^8^ In this population, the addition of targeted agents such as ibrutinib failed to show superiority over R-CHOP alone and even worsened progression free survival and overall survival due to excess toxicity and the lower proportion of patients receiving at least six cycles of R-CHOP (73.7% vs. 88.8% in the younger population in the PHOENIX trial).^9^ Of note, in 2022, after the end of the present study recruitment, the POLARIX study showed a benefice of the R-CHOP and polatuzumab vedotin combination as compared to R-CHOP, with a benefice mainly observed within patients with an ABC DLBCL.

Epigenetic modulation of histones plays a critical role in oncogenic transformation in many malignancies. Next-generation sequencing of B-cell NHL genomes uncovered frequent mutations affecting histone-modifying proteins, especially in lymphomas derived from the germinal center.^1^ EZH2 is the catalytic subunit of the chromatin remodeling polycomb repressive complex 2 (PRC2) and a unique human protein methyltransferase able to methylate histone 3 lysine 27 (H3K27), leading to the transcriptional repressive mark H3K27me3. Indeed, it impacts hematopoiesis and germinal center development by repressing genes involved in cell cycle arrest and terminal differentiation^10^ as opposed to the switch/sucrose non-fermentable (SWI/SNF) chromatin-remodeling multiprotein complex. Disrupting this differentiation process, gain-of-function mutations in *EZH2* and loss-of-function aberrations in the SWI/SNF complex (including *ARID1A*, *PBRM1*, and *SMARCC2* mutations) are recurrent abnormalities in DLBCL, resulting in an aberrant proliferative dependency on EZH2 activity.^11^^,^^12^ Beside this conventional function, EZH2 can also plays an important and pleiotropic role in a range of biological processes, though the methylation of non-histone proteins and/or methyltransferase independent mechanisms modulating gene-expression programs,^13^ or physical interactions with DNA-binding factors and transcriptional coactivators.

Tazemetostat (TAZ) is a selective oral EZH2 inhibitor which has US Food and Drug Administration approval in follicular lymphoma.^2^ In DLBCL, it showed a favorable safety profile and activity in patients with *EZH2* wild-type (WT) or mutant tumors,^3^ likely due to its pleiotropic functions. Given the favorable safety profile of TAZ as a single agent, we conducted a phase Ib/II study Epi-R-CHOP (NCT02889523) to explore the safety and activity of combining TAZ with R-CHOP in elderly patients newly diagnosed with DLBCL. In phase I of the trial, we reported that R-CHOP plus TAZ was well tolerated with safety and pharmacokinetic results comparable to R-CHOP alone, and the recommended phase II dose of TAZ in combination with R-CHOP was consistent with TAZ monotherapy (800 mg BID). We report here the efficacy results of phase II of the TAZ R-CHOP study conducted in patients aged 60–80 years with newly diagnosed DLBCL.

## Methods

### Study design

We report here the phase II part of this open-label, multi-center study of tazemetostat (EPZ-6438) in combination with standard doses of intravenous rituximab plus CHOP in elderly DLBCL patients (ClinicalTrials.gov identifier: NCT02889523, Eudract: 2016-001499-31). The study protocol was approved by the institutional review board and ethics committees at participating institutions in accordance with the International Conference on Harmonization guidelines, including good clinical practice and ethical principles based on the Declaration of Helsinki. The recommended phase II dose was identified in the phase Ib dose escalation part at 800 mg BID, without observing dose limiting toxicity.^14^

#### Ethics approval and consent to participate

The study protocol was approved by the institutional review board and ethics committees at participating institutions in accordance with the International Conference on Harmonization guidelines, including good clinical practice and ethical principles based on the Declaration of Helsinki. Consent for publication: all the patients provided their informed consent.

Patients were recruited in 27 LYSA centers in France and Belgium from July 31, 2020 to July 18, 2022.

### Treatment

Patients received up to six cycles of standard R-CHOP regimen every 21 days (rituximab 375 mg/m^2^ D1, prednisolone oral 40 mg/m^2^ D1-5, doxorubicine 50 mg/m^2^ D1, cyclophosphamide 750 mg/m^2^ D1, vincristine 1.4 mg/m^2^ D1) in combination with continuous tazemetostat at 800 mg BID starting on D2 of R-CHOP cycle 1 (C1), followed by two cycles of tazemetostat and rituximab (i.e., cycles 7 and 8). Prophylaxis with granulocyte colony-stimulating factors was used to prevent febrile neutropenia according to the recommendations of the American Society of Clinical Oncology.^15^ Valacyclovir and cotrimoxazole prophylaxis were mandatory. Dose adaptation was permitted. In this elderly population, following a safety alert regarding constipation AE, after the inclusion of the first 24 patients in the phase II part, it was decided to reduce the dose of vincristine to 1 mg total dose. A full list of the dose adaptation rules is provided in the Supplementary Material.

### Inclusion and exclusion criteria

Eligible patients were aged between 60 and 80 years with a diagnosis of untreated DLBCL: age-adjusted International Prognostic Index (aaIPI) 1+ or International Prognostic Index (IPI) 2+. Other eligibility criteria were as follows: ECOG performance status of 0–2, adequate renal function (creatinine clearance > 40 mL/min), bone marrow function (ANC ≥ 1500/mm^3^, platelets ≥ 75,000/mm^3^, hemoglobin ≥ 9 g/dL), adequate liver function, and left ventricular ejection fraction >50%. Patients were excluded if they presented central nervous system or meningeal involvement, had received prior treatment with any EZH2 inhibitor or any previous lymphoma treatment, or had any conditions that might compromise their safety during the study according to the investigator. A full list of the inclusion and exclusion criteria is provided in the Supplementary Material.

### Endpoints and assessments

Primary endpoint was positron emission tomography complete metabolic response rate (CMR) (i.e., Deauville score 1–3, Cheson criteria, Lugano 2014, local assessment) at the end of treatment or at the time of premature treatment discontinuation (PTD). Data cut-off was January 30, 2023.

Key secondary objectives were progression free survival (PFS), duration of response (DOR), and overall survival (OS). Time-to-event survival curves were estimated using the Kaplan–Meier method. PFS was defined as the time from study inclusion to the first observation of documented disease progression or death due to any cause. In the absence of progressive disease or death, PFS was based on an adequate assessment at the time of the last follow-up visit. OS was measured from the date of inclusion to the date of death from any cause. Alive patients were censored at the last follow-up date.

### Statistical analysis

Sample size was calculated with the expectation of a 10% increase in CMR, with an H0 hypothesis of 70%^9^^,^^16^^,^^17^ and an H1 assumption of 80%, leading to a sample size of 115 treated patients and 122 included patients, assuming a drop-out rate of 5%, α risk of 0.05, and β of 0.20 (one-sided). Patients without a response assessment (irrespective of the reason) were considered non-responders. Interim analysis for futility was scheduled after the inclusion of 60 patients, requiring at least 41 CMR to reject the null hypothesis (i.e., futility defined as less than 40 CMR or 67%). This analysis was positive with 42 patients that reached a CMR (70%), 5 a PMR, 2 a progressive disease and 11 that were not evaluated (18.3%). This allowed the continuation of the study and conduction of the second stage analysis, reported here.

The **efficacy and safety set** corresponds to patients who signed informed consent and received at least one dose of TAZ. Initial sensitivity analysis was performed after excluding patients who withdrew consent (N = 10) (Supplementary Figure S1). Statistical analyses were conducted using SAS® version 9.3.

For time-to-event analyses, log-rank test were used to compare the survival distribution from two independent groups. Cox proportional hazard model were run after checking the assumption of proportional hazards. For continuous variables, Wilcoxon rank-sum test, also called Mann–Whitney U test, were used to compare distributions from two independent groups. For categorical variables, Chi2 Test were used to determine whether there is a significant association between variables.

### Pathological and molecular biomarker analysis

All but one diagnosis was reviewed centrally, with tumors being classified according to the Hans algorithm. These exploratory analyses were performed for the **sensitivity set** (N = 112 patients), excluding cases with a centrally reviewed diagnosis that did not correspond to DLBCL (N = 4 follicular lymphoma grade 3A).

We assessed the association between the primary endpoint (i.e., CMR) on the one hand, and *EZH2* mutational status, genomic classification, immunohistochemistry methylation score, and gene expression-based cell of origin on the other. *EZH2* mutational status was assessed at diagnosis using cfDNA and/or formalin-fixed paraffin-embedded (FFPE) tumor biopsy. Nucleic acid extraction was performed on the FFPE samples according to the standard procedure and on plasma samples using the QIAmp Circulating Nucleic Acid Kit (Qiagen). Gene expression-based cell of origin was assessed by reverse transcription and multiplex ligation-dependent probe amplification, and sequencing was performed with an amplicon-based technique as previously published.^18^ A targeted custom DNA panel including 47 lymphoma related genes (full or partial coding sequence, *PIM1, ARID1A, B2M, CCND3, CD58, CDKN2A, CIITA, CREBBP, CXCR4, EP300, FOXO1, GNA13, ID3, IRF4, MEF2B, PRDM1, SOCS1, TNFAIP3, TNFRSF14, TP53, CD70, DTX1, BCL10, KLF2, UBE2A, BTG1, SGK1, TET2, DUSP2, NFKBIA, ZFP36L1, STAT6, BRAF, BTK, CARD11, CD79A, CD79B, EZH2, MYD88, NOTCH1, NOTCH2, PLCG2, TCF3, XPO1, BCL2, CDKN2B, MYC*) was used to investigate LymphGen classification using methods described by Wright et al.^19^ Libraries were performed using 30 ng of tumor DNA and/or 30–60 ng of cfDNA, with an amplicon-based technique (QIAseq Targeted DNA Custom Panel, Qiagen), as previously described^20^ and sequenced on an Illumina device (NovaSeq, Illumina). EZH2 mutational status was determined on exons 16 and 18, targeted by the sequencing panel Fluorescence in situ hybridization (FISH) for MYC, BCL2, BCL6, rearrangements was performed using break-apart probes (LSI MYC Dual Color Break Apart Rearrangement Probe, Vysis, ZytoLight ® SPEC BCL2 Dual Color Break Apart Probe, Zytovision, ZytoLight ® SPEC BCL6 Dual Color Break Apart Probe, Zytovision). Slides were scanned and analyzed using Scanner PathScan® Combi, Excilone, x40, Z stack and interpretation was performed by two observers using consensus eyeballing. A methylation score IHC score based on H3K27me2 and H3K27me3 expression was calculated, as previously published.^14^

### Role of the funding source

This study was sponsored under a grant from Ipsen. Ipsen had no input into the study design, analysis, or interpretation of results.

## Results

### Patient population

Among the 122 included patients who initiated the study treatment, median age was 70 years (60–80), and 57% were female. Overall, 12% had an ECOG performance status of 2, 70.5% Ann Arbor stage IV disease, 19.7% stage III, 66% elevated lactate dehydrogenase (LDH), and 73.8% IPI of 3–5 (Table 1). Central pathological review confirmed the diagnosis of DLBCL in 117 cases, of whom 48.7% had a non-germinal center B-cell (GCB) subtype and 51.3% a GCB subtype based on the Hans algorithm (Table 2). In four cases, the diagnosis was confirmed as follicular lymphoma grade 3A. Gene expression-based molecular classification was available for 86 patients, of whom 41.9% had an activated B cell DLBCL and 40.7% GCB DLBCL. For cases with available FISH status, 28 presented a *BCL2* rearrangement (29.0%), 33 *BCL6* (34.4%), and 12 *MYC* (11.8%). Six cases featured both *MYC* and *BCL2* rearrangements (6.2%).

**Table 1:** Baseline clinical characteristics of the 122 patients included in the Epi-RCHOP study.

Baseline characteristics	Safety set, N = 122, N (%)
Female	70 (57.4%)
Male	52 (42.6%)
Age, mean (SD)	69.5 (5.24)
ECOG performance status	
0–1	108 (89.5%)
2	14 (11.5%)
Ann Arbor stage	
I–II	12 (9.8%)
III–IV	110 (90.2%)
LDH > UNL	80 (65.6%)
B symptoms, yes	30 (24.6%)
Extranodal involvement, 2 or more sites	54 (44.3%)
International Prognostic Index	
0–2	32 (26.2%)
3–5	90 (73.8%)
Age-adjusted International Prognostic Index	
1	47 (38.5)
2	68 (55.7%)
3	7 (5.7%)
Anemia (yes)	49 (40.2%)
Albumin level < UNL	22 (20.4%)

**Table 2:** Baseline pathological and molecular profiling based on the central pathology review of 121 available cases.

Central pathological review	N = 121
Diffuse large B cell lymphoma (NOS)	103 (85.1%)
High grade B cell lymphoma (double or triple hit)	8 (6.5%)
Large B cell lymphoma with IRF4 rearrangement	1 (0.8%)
Follicular lymphoma grade 3B	2 (1.7%)
T-cell-rich diffuse large B cell lymphoma	2 (1.7%)
Primary mediastinal b cell lymphoma	1 (0.8%)
Follicular lymphoma grade 3A	4 (3.3%)
Hans COO, missing N = 8 (6.6%)	N = 113
Germinal center	58 (51.3%)
Non-germinal center	55 (48.7%)
Gene expression classification,^a^ missing N = 35 (28.9%)	N = 86
Activated B cell	36 (41.9%)
Germinal center	35 (40.7%)
Other	15 (17.4%)
Missing	35
LymphGen (N = 76),^b^ missing N = 45 (37.2%)	N = 76
EZB	21 (27.8%)
ST2	5 (6.6%)
MCD	12 (1.6%)
BN2	13 (1.7%)
Other	25 (32.9%)
*EZH2* mutational status (N = 119), missing N = 2 (1.6%)	18 (15.1%)
BCL2 rearrangement (N = 96), missing N = 25 (20.6%)	28 (29.2%)
BCL6 rearrangement (N = 96), missing N = 25 (20.6%)	33 (34.4%)
MYC rearrangement (N = 102), missing N = 19 (15.7%)	12 (11.8%)
Double hit, MYC, and BCL2 (N = 96), missing N = 25 (20.6%)	6 (6.2%)

NOS: not otherwise specified; COO: cell of origin.

a Based on Bobée et al.^18^

b Based on Wright et al.^19^

### Treatment received

**Fig. 1:**
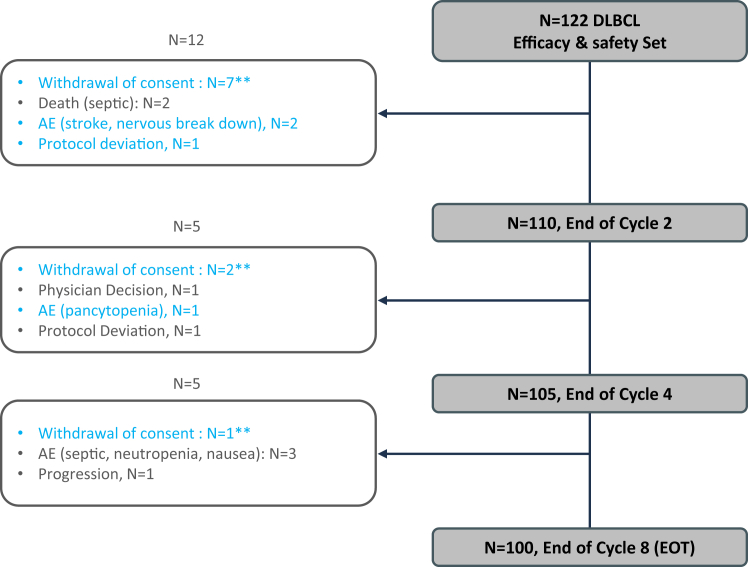
Patient flow chart. EOT: end of treatment; DLBLC: diffuse large B cell lymphoma; AE: adverse events; BM: bone marrow. ∗∗excluded from sensitivity analysis. Blue: patients not evaluated for response and considered non-responders.

A total of 100 patients (82%) received the planned eight treatment cycles, while 22 had a PTD (Fig. 1). Of the 22 cases of PTD, 12 occurred during cycles 1 and 2, and ten between cycles 3 and 8. The reasons for PTD were as follows: consent withdrawal (N = 10), adverse events (AE) (N = 6, including two during cycles 1 and 2), death (N = 2, both due to septic shock, namely *Klebsiella pneumonia* and an undetermined organism, during cycle 1 and considered treatment-related), protocol deviation (N = 2), progressive disease (N = 1), or physician decision (N = 1). The high number of consent withdrawal (N = 10, none of them being related to toxicity or early progression), especially in the early phase (7 patients during Cycle 1 and 2), was mainly triggered by 2 factors in this elderly patient population. First, seven of the ten patients complained it was too difficult to swallow eight TAZ pills twice a day in addition to other medications. Second, three out of the ten patients withdrew consent just after being informed of an external safety alert issued during treatment period and dealing with occurrence of acute leukemia in other TAZ trials. The six AE leading to PTD (i.e., discontinuation of TAZ and R-CHOP) in 4.9% of patients were one cerebrovascular event, depression, aplasia, septic thrombophlebitis, neutropenia, and nausea, four of which were considered to be treatment-related by the investigator. Six cycles of R-CHOP were administered to 84.4% of patients (N = 103), with a median time of 21 days (mean 21.5, Q1–Q3: 21–22) between cycles. In October 2020, after the inclusion of 24 patients, the total dose of vincristine was capped at 1 mg due to excessive constipation, despite appropriate supportive care (instead of 2 mg previously). The mean and median relative dose intensity for doxorubicin, cyclophosphamide, and vincristine exceeded 90% (Supplementary Table S1).

Overall, the median percentage of the planned TAZ dose received by patients was 98.4% (mean 77.2%, Q1: 61%; Q3: 100%), with 67.2% of patients receiving 75% or more of the planned dose and 79.5% receiving more than 50%. Additionally to the 22 patients that had a PTD, 10 (8%) permanently discontinued TAZ without stopping R-CHOP. Nine of these cases were due to AE (N = 4 nausea; N = 2 anemia, N = 1 for each of dysphagia, diarrhea, and thrombocytopenia), and four occurred before the end of cycle 3. Temporary dose interruption was observed in 80 patients (65.6%) due to an oversight in 76.6% or AE in 45.7% (considering that interruptions for a single patient can have several causes; Supplementary Table S2). From cycle 1 to cycle 8, we observed a reduction in both the overall incidence of interruptions and AE-related interruptions. Dose modifications due to AE occurred in 27 patients.

### Efficacy

#### Primary endpoint

**Fig. 2:**
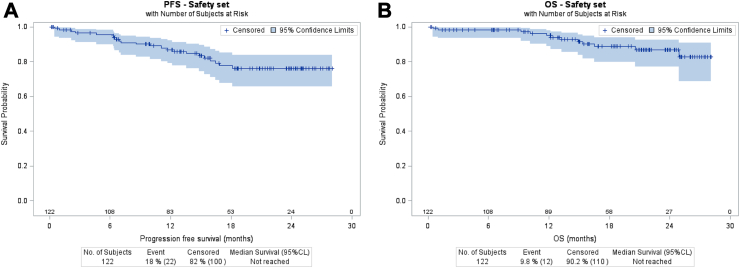
Progression free survival (A) and overall survival (B) since treatment initiation in the safety set of 122 patients.

At end of treatment or PTD, 92/122 patients (75.4%, 90% CI: 68.2–81.7%) achieved CMR, eight (6.6%) partial metabolic response (PMR), five (4.1%) had progressive disease, 2 (1.6%) died (septic shock during cycle 1), and 15 (12.3%) were not evaluated (14 due to PTD, and 1 due to out of delay PET-CT (was in CMR) see Fig. 1) and considered non-responders (Table 3). The primary objective was not met in this efficacy set given the CMR rate of 75.4% (less than the H1 of 80%) but in the sensitivity analysis, which excluded ten patients who had withdrawn consent (all non-evaluated; see Fig. 1), the CMR rate was 82.1% (92/112, 90% CI: 75.1–87.8%, power 0.77) and PMR rate 7.1%; five patients were not evaluated (and were considered as non-CMR). After a median follow-up of 18.5 months (range 0.2; 28.1; IQR (15.4–21)), 17/122 patients had disease relapse or progression (13.9%) and 12 had died: four from lymphoma, two from COVID-19, one from acute respiratory distress syndrome, four from AE (two septic shock, one acute myeloid leukemia after cycle 5, and one heart failure), and one unknown cause (in CMR). The estimated PFS and OS at 18 months were 77.7% (95% CI: 67.5–85.1%) and 88.8% (95% CI: 79.9–93.9%), respectively (Fig. 2A and B).

**Table 3:** Response rate and primary endpoint analysis.

Analysis sets	Safety set, N = 122	Sensitivity set,^b^ N = 112
Overall response rate	100 (82.0%)	100 (89.3%)
Complete metabolic response	92 (75.4%)	92 (82.1%)
Partial metabolic response	8 (6.6%)	8 (7.1%)
Progressive disease	5 (4.1%)	5 (4.5%)
Death	2 (1.6%)	2 (1.8%)
Not evaluated^a^	15 (12.3%)	5 (4.5%)

a Patients not evaluated at the end of treatment were considered non-metabolic response.

b Sensitivity set excludes the ten patients who withdrew consent and had no treatment evaluation.

#### Biomarker of response

Biomarker analyses were performed on the sensitivity cohort of 109 patients with a confirmed diagnosis of DLBCL (i.e., excluding patients with follicular lymphoma grade 3A after central pathological review and those who withdrew consent without undergoing a response assessment). *EZH2* mutational status was available for 107 patients using cfDNA at diagnosis, of whom 15 (14%) had a mutation (Supplementary Table S3). Genomic data and molecular classification based on the LymphGen classifier was available for 69 cases: 18 were classified as EZB, 12 as BN2, 11 as MCD, and four as ST2, while 24 were not classified. Using FISH, 23 patients had a *BCL2* rearrangement, 28 *BCL6*, 11 *MYC*, and seven presented double/triple-hit lymphomas (7.7%). Correlation between mutational status and other biological parameters (Supplementary Table S4) confirmed a significant correlation between *EZH2* mutated cases and GCB-subtypes. To avoid confounders in the outcome and biomarker analysis, we first analyzed the outcome of patients with *MYC/BCL2* and/or *BCL6* double-hit lymphoma (N = 7). Patients with double-hit lymphoma had a trend towards a lower CMR rate (57.1% vs. 85.7%, p = 0.085) and a shorter PFS compared with those without double-hit lymphoma (Supplementary Figure S2A), HR 5.722 (95% CI: 1.828–17.910, even though the wide 95% CI suggests an imprecise HR estimate). When excluding these high-grade double-hit lymphomas (N = 7) from the analysis, to be in accordance with the new WHO classification, there was no significant correlation between CMR or PFS/OS and cell of origin as assessed by the Hans algorithm, *EZH2* mutational status, methylation score, or LymphGen classification (Supplementary Table S5, Supplementary Figure S2B).

#### Safety

Within the safety set (N = 122 patients), the most frequent AE were neutropenia (53%), anemia (51%), nausea (47%), asthenia (42%), peripheral neuropathy (37%), gastrointestinal hypomotility (35%), weight loss (28%), vomiting (24%), and thrombocytopenia (24%) (Table 4). AE of grade 3 or more occurred in 73% of patients and were mainly hematological AE with neutropenia (48%, including 6% of febrile neutropenia), anemia (23%), thrombocytopenia (17%), and gastrointestinal disorder (10.7%). A total of 31 patients presented at least one serious AE (SAE) (25%), the most frequent being infection (3%) or gastrointestinal hypomotility (3%). Red blood cell and platelet transfusions were administered in 31% and 11.5% of patients, respectively. Overall, infection occurred in 38 patients (31%), including grade 3 or more in nine patients (7.4%). Among all AE, 4.1% led to TAZ interruption, 2.8% to TAZ discontinuation, and 2.4% to TAZ dose reduction. R-CHOP and TAZ were permanently discontinued due to AE in 8 cases (6.6%), including two cases of grade 5 (septic shock). In October 2020, after the inclusion of 24 patients, an alert was issued due to excess constipation, ileus, or fecaloma (constipation in 50% of patients, with 15 AE including two cases of grade 3 constipation). After reducing the dosage of vincristine, the incidence of these AE diminished substantially, with constipation-related AE subsequently observed in only 33 out of 122 patients (27%, with 43 AE, including one case of grade 3). Details are presented in Supplementary Table S6.

**Table 4:** Safety data in the phase II part of EpiCRHOP (reported here all grade >10%).

	Safety set, N = 122			
	All grades Number (%) patients with adverse event		Grade ≥3 Number (%) patients	
**Adverse event**	**119**	**(97.5%)**	**89**	**(73.0%)**
Neutropenia	65	(53.3%)	59	(48.4%)
Anemia	62	(50.8%)	28	(23.0%)
Nausea	58	(47.5%)	4	(3.3%)
Leukopenia	55	(45.1%)	49	(40.2%)
Asthenia	52	(42.6%)	1	(0.8%)
Neuropathy peripheral	45	(36.9%)	1	(0.8%)
Gastrointestinal hypomotility	43	(35.2%)	4	(3.3%)
Infection	38	(31.1%)	9	(7.4%)
Weight fluctuation	34	(27.9%)	5	(4.1%)
Thrombocytopenia	29	(23.8%)	21	(17.2%)
Vomiting	29	(23.8%)	3	(2.5%)
Pain	24	(19.7%)	0	(0.0%)
Stomatitis	24	(19.7%)	1	(0.8%)
Decreased appetite	19	(15.6%)	2	(1.6%)
Musculoskeletal pain	18	(14.8%)	2	(1.6%)
Diarrhea	15	(12.3%)	2	(1.6%)

## Discussion

EpiRCHOP was a phase I/II study that for the first-time combined TAZ, the first-in-class EZH2 inhibitor, with R-CHOP in untreated elderly patients newly diagnosed with DLBCL. The trial population was particularly challenging, with a median age of 70 years and high-risk baseline characteristics with anemia in 40%, elevated LDH in 66% and IPI of 3–5 in 74% of patients. In the intent-to-treat population, the primary endpoint was not met, with CMR of 75.4%. However, in per-protocol sensitivity analysis, the CMR reached 82.1%, suggesting an efficacy signal. Importantly, the majority (84.4%) of patients received six cycles of TAZ and R-CHOP with a median of 21 days between cycles and a relative dose intensity of R-CHOP agents greater than 90%, thus confirming that the drug combination is feasible.

Though acceptable, the safety profile of the combination led to constipation when the vincristine dosage was at 1.4 mg/m^2^. This issue was resolved by capping vincristine at a total dose of 1 mg, as usually given to elderly patients receiving R-CHOP. Regarding hematological toxicity, the incidence of grade 3 or more anemia (23%) was higher than expected with R-CHOP alone (7–8% in POLARIX or GOYA trials), although it was still in the range of other R-CHOP-X studies, showing an incidence between 10 and 35%.^9^^,^^17^^,^^21^, ^22^, ^23^, ^24^, ^25^, ^26^, ^27^, ^28^ The presence of anemia in 40% of patients at the time of diagnosis might have also impacted this incidence. Importantly, the incidence of grade 3 or more neutropenia (48.5%) was in line with data from the literature, although we observed a very low rate of febrile neutropenia (6%), which did not exceed that observed with R-CHOP(17) alone and was less than that found in R-CHOP-X studies^9^^,^^17^^,^^21^^,^^27^ (up to 20–33% with the novel combination^24^^,^^28^). We report two infection-related deaths during the treatment period (1.6%), which is similar or lower than R-CHOP alone.^17^ Indeed, in the original R-CHOP phase III trial, including the same elderly population, 16/399 (4%) infection-related deaths were reported.^29^ Finally, the incidence of grade 3 or more thrombocytopenia was slightly higher than that reported with R-CHOP alone,^9^^,^^17^^,^^21^ but remained manageable with only 11% of transfusions and in the lower range of R-CHOP-X combinations.^24^^,^^25^^,^^28^ At time of data analysis (median FU 18.5 months), one acute leukemia was reported, after 5 cycles of R-CHOP-TAZ, and no secondary myelodysplasia. Longer FU is however needed to fully assess this risk. Given the RDI of R-CHOP greater than 90%, considering the reduced dosage of vincristine at 1 mg, and the administration of the first six cycles in 84% of patients, this regimen appears feasible, unlike other R-CHOP-X combinations whose toxicity led to a significant reduction in dose intensity with a poorer outcome for elderly patients.^9^ Indeed, in EpiRCHOP, 6.5% of patients discontinued both R-CHOP and TAZ due to AE, which is much lower than the 35% reported in the elderly population of a recent ibrutinib-R-CHOP trial.^9^

Regarding efficacy, when analyzed as per protocol, our data show a CMR rate of 82%, which meets our initial assumption. Cross-trial comparison is not feasible. Even though the population of patients included in EpiRCHOP was older and had a greater incidence of intermediate to high risk IPI (70%), the results presented here seem to be more favorable compared with other R-CHOP-X studies (R-CHOP-ibrutinib,^9^ R-CHOP-venetoclax,^28^ or R-CHOP-polatuzumab^17^) and approach the experimental arm of the positive GUIDANCE study (88% of CMR)^21^ or another combination with hypomethylating agents (Aza-RCHOP^30^). An R-CHOP comparison arm is needed to properly interpret the biomarker analysis. However, the absence of correlation between *EZH2* mutational profile or LymphGen classification subgroups (EZB vs. other) on the one hand and CMR on the other suggests that TAZ has a broader mechanism of action and/or that a distinct molecular alteration can lead to similar functional consequences as the *EZH2* gain-of-function mutation, as reported in follicular lymphoma.^31^ Our translational analysis however has limitations due to the missing data inducing selection biais. The combination of TAZ and R-CHOP might also improve the poor chemosensitivity observed in these tumors, as shown in the GUIDANCE trial.^21^ Given the potential prolonged effect of TAZ on epigenetic modulation, additional analysis as well as a longer follow-up are both needed for the identification of relevant biomarker of response duration. This longer follow-up will also help to precise the future of TAZ-RCHOP combination in the actual moving field of first line DLBCL. Indeed, the combination appears feasible at the toxicity level, but the prolonged benefice/risk of an epigenetic modulation in first line requires a precision medicine approach. We acknowledge some limitations as sparse-data cells for correlative biological analysis.

The combination of TAZ and R-CHOP is feasible and leads to promising CMR in an elderly population of patients with DLBCL. Longer follow-up is needed to assess if the combination impacts the survival outcome with a potential prolonged imprint of this epigenetic treatment on the duration of response in specific molecular subgroups.

## Contributors

CS, VR, and CP accessed and verified the data: CS, LY, FM, and VR designed the clinical trial and wrote the manuscript. CP performed statistical analysis. TM and LV performed the central pathology review. FJ, SD, EB, and PR performed the translational analysis. LY, RH, GMP, CH, TG, CH, SG, CB, GC, CT, SC, EB, FJ, FM, and VR included patients in the study. All the authors reviewed the manuscript.

## Data sharing statement

Data will be shared based on LYSA/LYSARC policy. For further information, please consult the PDF provided.

## Declaration of interests

C.S: Advisory Board: Janssen, Beigene, Abbvie, Roche, MSD; Honoraria: Astra-Zeneca, Beigene, Incyte, Roche, MSD; Congress fees: Roche, GILEAD, Takeda, Abbvie; Research fundings: Roche. C.T: Payment or honoraria for lectures, presentations, speakers bureaus, manuscript writing or educational events, consulting, advisory board: Kyte/Gilead, BMS, Novartis, Roche, Incyte, Abbvie, AstraZeneca; Support for attending meetings and/or travel: Abbvie, BMS, Novartis, Roche, Beigene. C.H: Honoraria: Roche, Janssen-Cilag, Abbvie, Incyte; Research Funding: Takeda; Travel, Accommodations, Expenses: Janssen-Cilag, Abbvie, Incyte. L.Y: Abbvie, AstraZeneca, Beigene, BMS/Celgene, Gilead/kite, Janssen, Roche. R.H.: honoraria from Kite/Gilead, Novartis, Incyte, Janssen, MSD, Takeda, Amgen, Abbvie and Roche; and is a member on an entity's Board of Directors or advisory committees of Kite/Gilead, Novartis, Bristol-Myers Squibb/Celgene, Incyte, Roche and Miltenyi. C.B: no COI; G.C: COI: honorary/travel grant from Gilead, Roche, Sobi, and AbbVie.; E.B: no COI; G.M.P: no COI; P.R: no COI; S.D: no COI; L.R: no COI; V.R: Advisory Board: Abbvie, Astex, Astra-Zeneca, Beigene, Ipsen, Lilly; Research funding: GSK; Pegascy: Employment, ; T.M: no COI; E.B: AbbVie, Roche, Takeda: advisory committees; ADC Therapeutics, BeiGene, Bristol Myers Squibb, Incyte, Novartis, Amgen, Kite,Pfizer: Honoraria: Janssen; Research Funding Amgen; F.M: consultancy: Roche/Genentech; consultancy and membership on an entity's Board of Directors or advisory committees: Kite/Gilead Sciences, Bristol Myers Squibb, AbbVie, Epizyme, AstraZeneca, Novartis, Genmab; honoraria: Roche/Genentech, Chugai, Takeda; S.C: honoraria from Abbvie, Takeda, Janssen, Novartis, Gielad, Pierre Fabre, BMS, Astra Zeneca, Amgen, Lilly; C.H: Honoraria from Abbvie, BMS, Incyte, Kite Gilead, Miltenyi, MSD, Roche, Sobi & Takeda; F.J: no COI; T.G: no COI; S.G: no COI.
