# Supplementary material for: Efficacy of tazemetostat in combination with R-CHOP in elderly patients newly diagnosed with diffuse large B cell lymphoma: results of the EpiRCHOP phase II study of the LYSA
Source: eClinicalMedicine. 2025 Mar 18;82:103157. doi: 10.1016/j.eclinm.2025.103157 (PMC11957796; doi:10.1016/j.eclinm.2025.103157)
Supplement: Supplementary Figures S1 and S2 [file mmc1.pptx]

## Slide 1
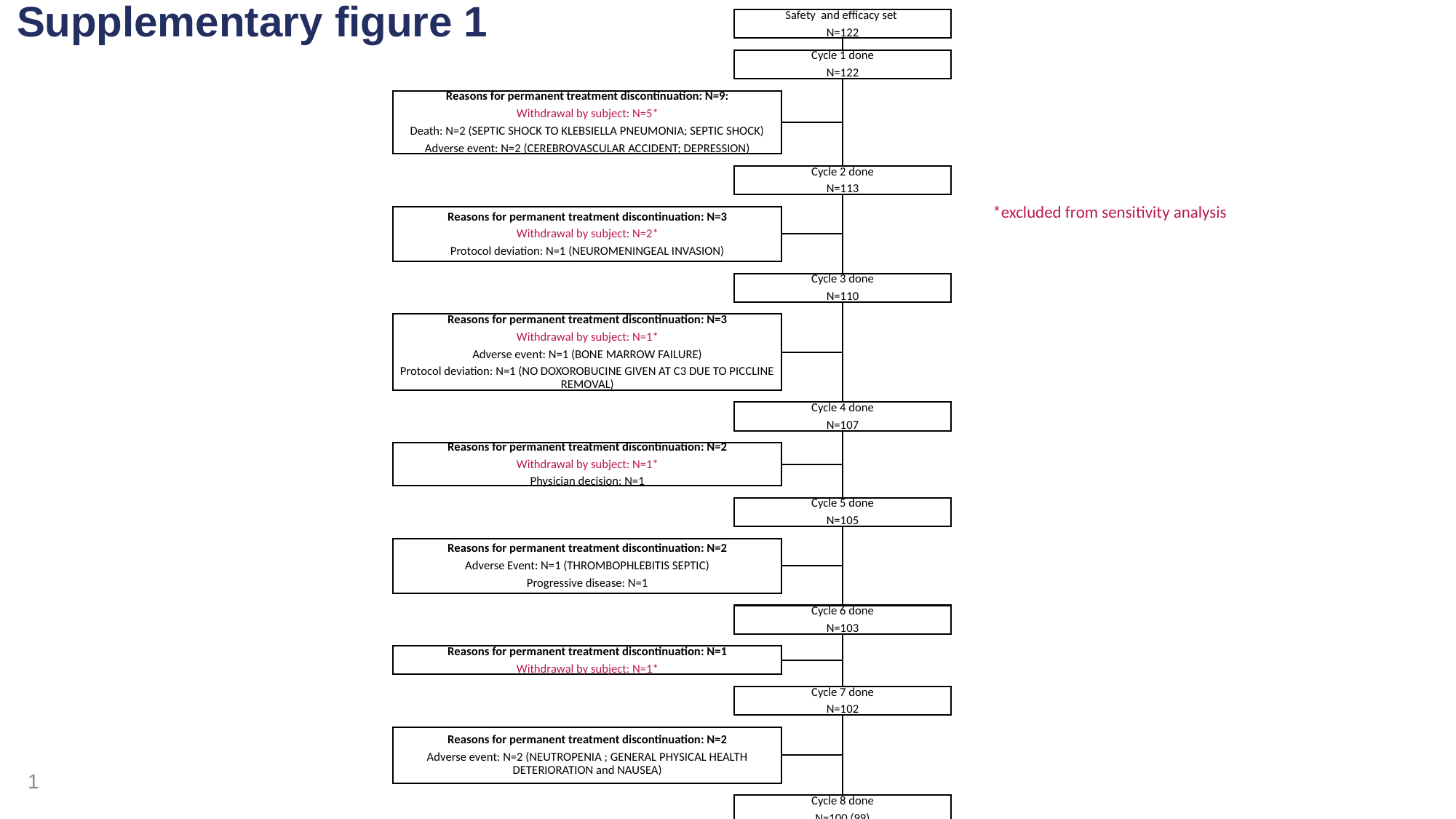

# Supplementary figure 1
*excluded from sensitivity analysis
1

## Slide 2
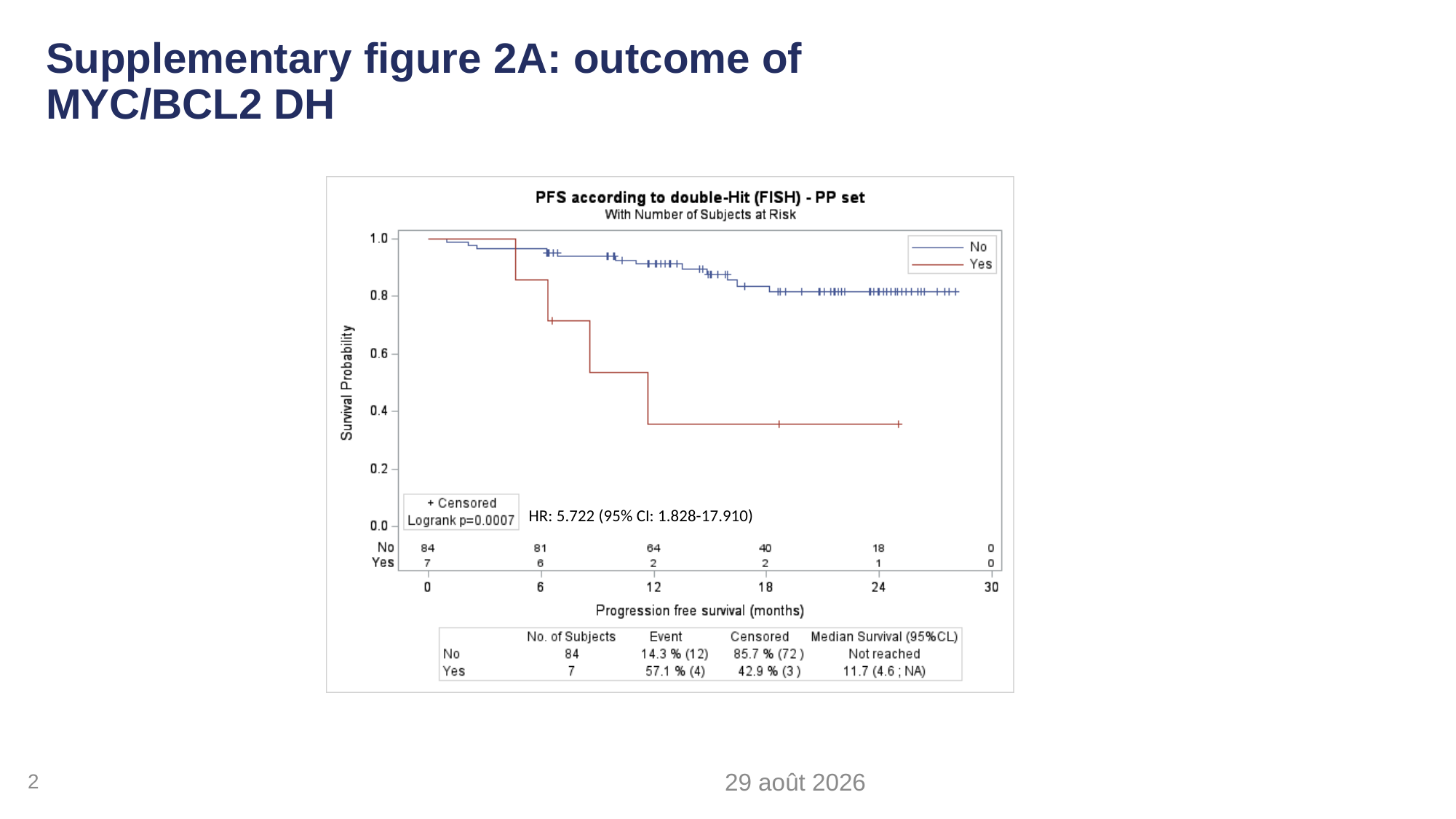

# Supplementary figure 2A: outcome of MYC/BCL2 DH
HR: 5.722 (95% CI: 1.828-17.910)
2
5 mars 2025

## Slide 3
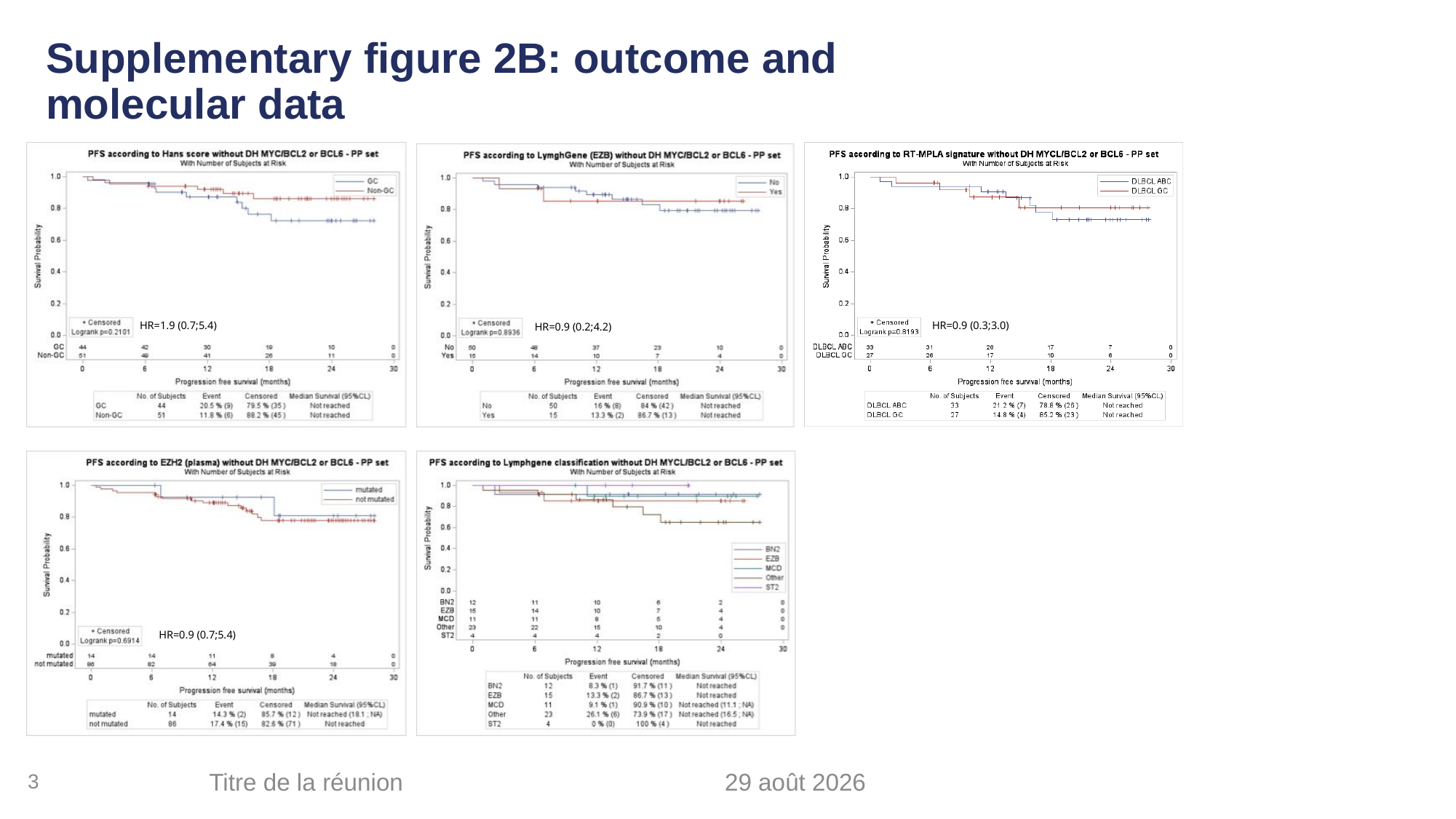

# Supplementary figure 2B: outcome and molecular data
HR=1.9 (0.7;5.4)
HR=0.9 (0.3;3.0)
HR=0.9 (0.2;4.2)
HR=0.9 (0.7;5.4)
3
Titre de la réunion
5 mars 2025
